# Possible interspecific origin of the B chromosome of *Hypsiboas albopunctatus* (Spix, 1824) (Anura, Hylidae), revealed by microdissection, chromosome painting, and reverse hybridisation

**DOI:** 10.3897/CompCytogen.v8i3.7771

**Published:** 2014-08-08

**Authors:** Simone Lilian Gruber, Débora Diniz, Patrícia Elda Sobrinho-Scudeler, Fausto Foresti, Célio Fernando Baptista Haddad, Sanae Kasahara

**Affiliations:** 1 UNESP, Universidade Estadual Paulista, Instituto de Biociências, Departamento de Biologia, Av. 24A 1515, 13506-900, Rio Claro, SP, Brazil; 2 UESB, Universidade Estadual do Sudoeste da Bahia, Departamento de Ciências Biológicas, Rua José Moreira Sobrinho s/n, Jequiezinho, 45206-190, Jequié, BA, Brazil; 3 UNESP, Universidade Estadual Paulista, Instituto de Biociências, Departamento de Morfologia, Distrito de Rubião Júnior s/n, 18618-970, Botucatu, SP, Brazil; 4 UNESP, Universidade Estadual Paulista, Instituto de Biociências, Departamento de Zoologia, Av. 24A 1515, 13506-900, Rio Claro, SP, Brazil

**Keywords:** Supernumerary chromosome, FISH, GISH, replication banding, Hylidae family, heterochromatin, repetitive DNA

## Abstract

The B chromosome in the hylid *Hypsiboas albopunctatus* (2n = 22 + B) is small, almost entirely composed of C-positive heterochromatin, and does not pair with any chromosome of the A complement. B probe, obtained by microdissection and DOP-PCR amplification, was used to search for homology between the B and regular chromosomes of *H. albopunctatus* and of the related species *H. raniceps* (Cope, 1862). Reverse hybridisation was also carried out in the investigation. The B probe exclusively painted the supernumerary, not hybridising any other chromosomes in *H. albopunctatus*, but all *H. raniceps* chromosomes showed small labelling signals. This result might be an indication that differences exist between the repetitive sequences of A and B chromosomes of *H. albopunctatus*, and that the chromosomes of *H. raniceps* and the heterochromatin of the B chromosome of *H. albopunctatus* are enriched with the same type of repetitive DNA. In meiotic preparations, the B labelled about 30% of scored spermatids, revealing a non-mendelian inheritance, and the painted B in micronucleus suggests that the supernumerary is eliminated from germ line cells. Although our results could suggest an interespecific origin of the B at first sight, further analysis on its repetitive sequences is still necessary. Nevertheless, the accumulation of repetitive sequences, detected in another species, even though closely related, remains an intriguing question.

## Introduction

B chromosomes are extra elements present in the genome of diverse groups of plants and animals (Jones and Rees 1982; Green 1990; Jones and Houben 2003; Camacho 2004, 2005; Schmid et al. 2010).With exception of dispensability for normal growth and development, many of the B characteristics, for example, numerical variability within and between individuals, smaller size than chromosomes from the A complement, heterochromatic nature, abnormal segregation at cell divisions, and non-mendelian inheritance, are not universal, indicating that the supernumerary chromosomes might correspond to distinct complex systems whose origins in the species are still the subject of extensive discussions.

To date, approximately 2% of karyotyped species of amphibians (Schmid et al. 2010; Green 2004) have shown B chromosomes, but among them only 16 anuran species were described with supernumeraries (Schmid et al. 2010; Green 2004; Milani et al. 2010). One of the most intriguing cases was reported in *Leiopelma hochstetteri* Fitzinger, 1861 from New Zealand, carrying two types of B chromosomes, one of them possibly related to a 0W/00 sex chromosomes system (Green 1988, 1991). Analyses on the molecular content of amphibian B chromosomes are scarce, and the only reported cases are in the frog *Leiopelma hochstetteri* (Sharbel et al. 1998) and in the salamander *Dicamptodon tenebrosus* (Baird & Girard, 1852) (Brinkman et al. 2000). In both cases, microdissected B chromosomes were analysed by Southern blotting, revealing that the B chromosomes share repetitive sequences with chromosomes of the A complement. The B chromosome of *Leiopelma hochstetteri* was also reported to contain specific sequences that were not present in any A chromosomes. Recently, B chromosomes of two species of Brazilian frogs, *Hypsiboas albopunctatus* and *Physalaemus feioi* Cassini, Cruz, & Caramaschi, 2010, were microdissected to generate probes for chromosome painting (Gruber et al. 2011; Campos et al. 2012).

Our previous analysis (Gruber et al. 2007) in the tree frog *Hypsiboas albopunctatus* had shown one B chromosome in some individuals. This supernumerary was very similar in size and morphology to NOR-bearing chromosome 8, and had a large amount of late replicating C-positive heterochromatin, with a small bright DAPI region in the short arms. It was observed that the B was univalent, not pairing with any other chromosome in metaphase I cells. The present work was carried out to investigate whether the B of *Hypsiboas albopunctatus* shares sequence homology with chromosomes of the A complement of the same species or of its closely related species *Hypsiboas raniceps* which could shed light upon questions about its intraspecific or interspecific origin. We used microdissection of B and chromosome painting (FISH), and additionally genomic hybridisation (GISH) with DNA of *Hypsiboas raniceps* in the metaphases of *Hypsiboas albopunctatus* harbouring one B chromosome.

### Material and methods

In this study, we used chromosome preparations from fourmales of *Hypsiboas albopunctatus*, among which three had B chromosome, and from two females of *Hypsiboas raniceps*:

*Hypsiboas albopunctatus*: São Paulo: Rio Claro (22°25'20"S, 47°34'23"W), CFBH 28554-57 (males).

*Hypsiboas raniceps*: Goiás: Ilha do Bananal (10°46'07.9"S, 50°00'12.1"W), CFBH 7431 (female). Mato Grosso: Santa Rita doTrivelato (13°47'10.2"S, 55°13'18.0"W), CFBH 22456 (female).

Voucher specimens were deposited in the amphibian collection Célio F.B. Haddad (CFBH) housed in the Departamento de Zoologia, Instituto de Biociências, UNESP, Rio Claro, SP, Brazil.

Direct cytological suspensions of bone marrow, liver, and testes were obtained according to Baldisera Jr. et al. (1993). The specimens of *Hypsiboas albopunctatus* were injected with 5-bromodoxiuridine (BrdU) before colchicine treatment (Silva et al. 2000). Slides were conventionally stained with Giemsa and those of *Hypsiboas albopunctatus* with B chromosome were also submitted to FPG (Fluorochrome Plus Giemsa) technique (Dutrillaux and Couturier 1981; Matsuda and Chapman 1995) to differentiate replication banding. The chromosomal images were captured with an Olympus digital camera D71 using the DP Controller program. Bi-armed chromosomes were classified as metacentric, submetacentric, or subtelocentric (Green and Sessions 1991, 2007).

Microdissections of B chromosome were carried out according to Dinizet al. (2008) from metaphase I cells of two male specimens of *Hypsiboas albopunctatus* with 2n = 22 + B. The specimens CFBH 28554 and CFBH 28557 were used to generate the B54 and B57 probes, respectively. A meiotic cell suspension was dropped onto a 24 mm × 60 mm coverslip and was immediately stained with 5% Giemsa in phosphate buffer, pH 6.8. Using a glass needle micromanipulator coupled in a Nikkon inverted microscope, four B chromosomes were microdissected and transferred to a microtube containing 10 μL DOP-PCR mix (1x Thermo Sequenase buffer reaction, 0.2 mMdNTP, 2 μM DOP primer - 5’ CCG ACT CGA GNN NNN NAT GTG G 3’ - (Telenius et al.1992), and ultra-pure water up to 10 μL). This procedure was repeated four times for each individual.

PCR reactions were performed using a Veritti Thermocycler (Applied Biosystems). Samples were heated at 90°C for 10 minutes and 4U. Thermo Sequenase enzyme (USB) was added. The initial amplification of microdissected products was performed using RAMP-PCR with the following conditions: 94°C for 3 min; followed by 12 cycles of 94°C for 1 min 30 s; 37°C for 2 min increasing 0.2°C/s up to 72°C; 72°C for 2 min; followed by another 30 cycles of 94°C for 1 min 30 s; 62°C for 1 min; 72°C for 1 min 30 s.

After RAMP-PCR, a standard PCR was carried out to generate a probe stock. This reaction was comprised of: 1x PCR buffer; 2 mM MgCl_2_; 0.02 mMdNTP; 0.7 mM DOP primer; 2.5 U Taq polymerase; 2 μL RAMP-PCR products; and ultra-pure water up to 25 μL. The following PCR conditions were used: 90°C for 3 min; followed by 30 cycles at 90°C for 1 min 30 s; 56°C for 1 min 30 s; 72°C for 1 min 30 s. Finally, a third PCR was performed to label the microdissection products. The PCR reaction was comprised of: 1× PCR buffer; 2 mM MgCl_2_; 0.05 mMdATP; 0.05 mMdCTP; 0.05 mMdGTP; 0.035 mMdTTP; 0.015 mM labelled dUTP (Digoxigenin-11-dUTP, Roche); 0.7 μM DOP primer; 2.5 U Taq polymerase; 3 μL of the products from the second PCR; and ultra-pure water up to 25 μL. The amplification steps were the same as described for the previous reaction. After each PCR, electrophoresis of the amplified samples was performed using a 1% agarose gel to verify the sizes of the fragments. The fragments were 400–800 bp. Each labelled PCR product was precipitated in ethanol and re-suspended in 10 25 μL.

The B54 and B57 probes were used to perform FISH in mitotic and meiotic chromosomes. Firstly, the probes were tested on samples from the individuals they were generated from, i.e., B54 was tested on cytological preparations from CFBH 28554 and probe B57, on preparations from CFBH 28557. Next, cross-specimen chromosome painting was performed in which the B54 probe was used on mitotic preparations from CFBH 28557. The B54 probe was also used on chromosome preparations of *Hypsiboas albopunctatus* specimen with no B chromosome (CFBH 28556), and on chromosome preparations of *Hypsiboas raniceps* specimens from two distinct localities (CFBH 7431 and CFBH 22456).

All FISH experiments were performed according to Pinkel et al. (1986) with modifications. The B probe hybridisation procedures were performed with 77% stringency to prevent non-specific labelling. For the reverse hybridisation, the probe obtained from the genomic DNA of *Hypsiboas raniceps* was marked with digoxigenin by nick translation and 50% stringency was used.

### List of Abbreviations

2n: diploid number; DAPI: 4′-6-diamidino-2-phenylindole; DOP-PCR: degenerate oligonucleotide-primed polymerase chain reaction; FISH: fluorescent *insitu* hybridisation; NOR: nucleolar organiser region; PCR: polymerase chain reaction.

## Results

**Figure 1.**
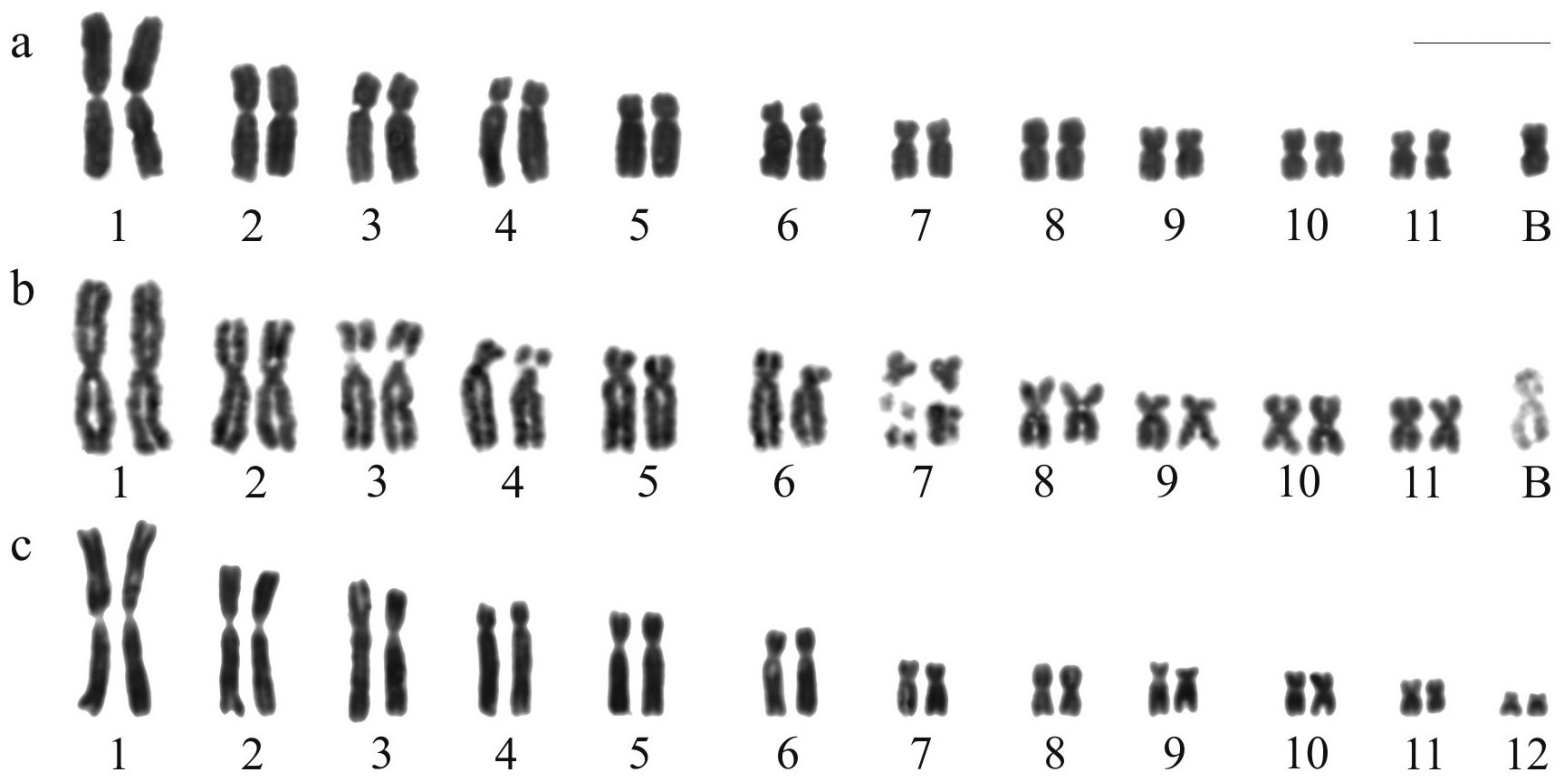
Karyotypes of *Hypsiboas*. **a–b** *Hypsiboas albopunctatus*, male, 2n = 22 + B **c** *Hypsiboas raniceps*, female, 2n = 24 **a, c** Giemsa stained karyotypes **b** replication bands. Bar = 10 mm.

Fig. 1a shows the karyotype of *Hypsiboas albopunctatus* with 2n = 22 + B chromosome after standard staining, and Fig. 1b, after FPG technique differentiating very late replicating regions in some chromosomes. The standard stained karyotype with 2n = 24 of *Hypsiboas raniceps* is presented in Fig. 1c.

**Figure 2.**
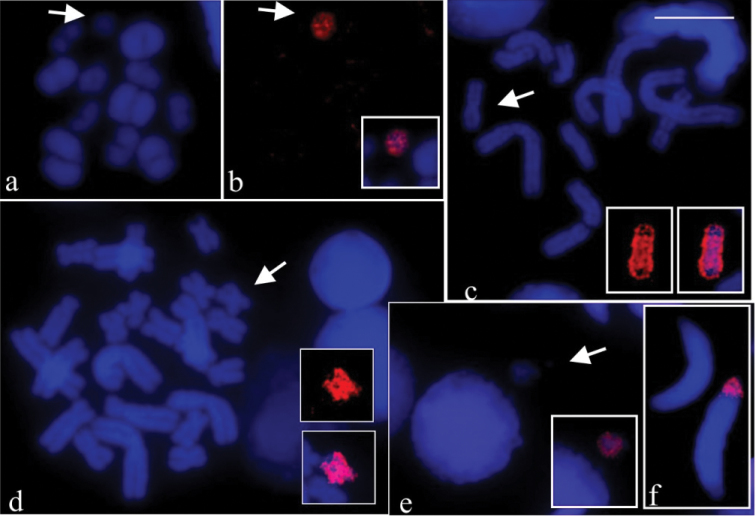
FISH using B probe in meiotic and mitotic preparations of *Hypsiboas albopunctatus*. **a–b, e–f** *Hypsiboas albopunctatus* (CFBH 28554), 2n = 22 + B; **c–d** *Hypsiboas albopunctatus* (CFBH 28557), 2n = 22 + B **a** DAPI stained metaphase I **b** the same cell of **a** showing the B54 probe painting and the merged image of the B (inset) **c** DAPI stained metaphase II and B chromosome of same cell of **c** showing the B57 probe painting (inset), and merged images of the B (inset) **d** DAPI stained mitotic metaphase and B chromosome of same cell of **d**, showing the B54 probe painting (inset), and merged images of the B (inset) **e** DAPI stained interphase nucleus and micronucleus, and merged images showing B probe hybridisation on micronucleus (inset) **f** spermatids with and without labelling of B probe. Note in **a**–**d** completely labelled B chromosome (arrows). Bar = 10 mm.

The probes B54 and B57 hybridised completely with the B in mitotic or meiotic preparations of the specimens they were generated from, i.e., the B54 probe painted the B chromosome in CFBH 28554 and the B57 probe painted the B chromosome in CFBH 28557. Fig. 2a shows a metaphase I cell of CFBH 28554 with DAPI staining and Fig. 2b the same cell with painted B54 probe. In Fig. 2c the painted B57 probe is shown in a metaphase II cell of CFBH 28557. Chromosomes of the A complement were not hybridised with any of the probes B54 and B57. The B54 probe cross-tested on mitotic preparation from the CFBH 28557 specimen also showed painting exclusively on the B chromosome (Fig. 2d). We performed a total of eight FISH experiments in which the B probes were tested on cells of individuals carrying the B chromosome, and identical results were always obtained. When the B54 probe was tested on mitotic preparation from *Hypsiboas albopunctatus* without B chromosome (CFBH 28556) no labelling was observed in the chromosomes (figure not shown). The B54 probe tested on the meiotic preparations of the *Hypsiboas albopunctatus* specimen CFBH 28554 with 2n = 22 + B showed that a micronucleus with the painted B probe was occasionally observed, close to an interphase nucleus (Fig. 2e). In the same cytological preparation, the B probe labelling was either present or absent in spermatids (Fig. 2f). The presence of B in spermatids was scored, showing B in 13% of the 66 analysed cells of one specimen, whereas in another, the B was observed in 31% of the 507 spermatids.

**Figure 3.**
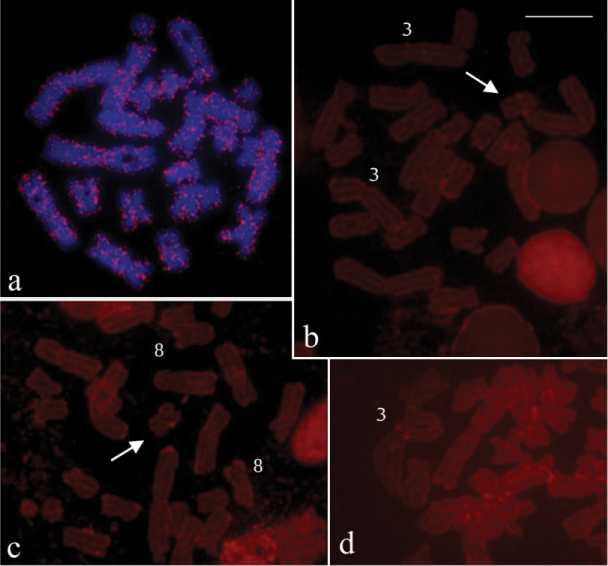
FISH using B probe in mitotic preparations of *Hypsiboas raniceps* and reverse hybridisation in *Hypsiboas albopunctatus*. **a** *Hypsiboas raniceps* (CFBH 7431), female, 2n=24 **b–c** *Hypsiboas albopunctatus*, male, 2n = 22 +B (CFBH28557) **d** *Hypsiboas albopunctatus*, male, 2n = 22 +B (CFBH28555) **a** chromosome painting with B54 probe, showing interspersed labelling in all chromosomes **b–d** reverse hybridisation withg gDNA of *Hypsiboas raniceps* probe, showing labelling on B chromosome (arrow) and on chromosomes 3 and 8. Bar = 10 mm.

The hybridisation experiment with B probe on preparations of two *Hypsiboas raniceps* specimens (CFBH 7431 and CFBH 22456), from different locations, showed numerous hybridisation signals interspersed throughout the chromosomes (Fig. 3a). The reverse hybridisation using gDNA of *Hypsiboas raniceps* showed a small hybridisation signal on the supernumerary of metaphases from *Hypsiboas albopunctatus* with B, and eventually, hybridisation signal was observed on some other chromosomes, like the 3 and 8 of A complement (Fig. 3b–d).

## Discussion

The A chromosome complement of *Hypsiboas albopunctatus* of the present study is in agreement with that previously reported (Cardoso 1986; Feitosa et al. 1995; Gruber et al. 2007; Ferro et al. 2012). One single B chromosome was observed in some of the Brazilian populations, whereas in Argentinian populations Ferro et al. (2012) identified two distinct morphological types of B, B1 and B2, with frequency varying from zero to three Bs per individual. The heterochromatin nature of the B was demonstrated by different cytogenetic techniques, including the here presented late replicating banding. The B chromosome appeared with negative staining in both proximal short and long arms, excepting the terminal ends, and this result is in accordance with the C-positive staining observed only in the proximal arms (Gruber et al. 2007).

To obtain the supernumerary probe of *Hypsiboas albopunctatus*, the microdissections performed in metaphase I cells, instead in mitotic metaphases, actually represented a good option. The univalent B was identified with certainty, preventing misidentification with the chromosomes 8. Besides, the smaller number of elements in the metaphase I meant better chromosome spreading and this avoided contamination during the scrapping procedure. The clear results of the FISH experiments indicate that the B chromosome probes were successfully generated, and the 77% of stringency prevented the probe hybridising non-specifically.

In all FISH experiments in which the B54 and B57 probes were tested in mitotic or meiotic cells of the specimens they were generated from, and in the case of cross-species, uniform and intense fluorescence was observed exclusively on the B chromosome. All these painting results are evidence that the B of *Hypsiboas albopunctatus* harbours a large amount of repetitive DNA sequences, a usual characteristic for the majority of the supernumeraries. Furthermore the identical results in the tests using probes obtained from different individuals indicate a significant degree of sequence conservation between the Bs in the studied population. The B probes did not paint any A chromosome in all experiments, including when tested in the *Hypsiboas albopunctatus* specimen without supernumerary. The lack of hybridisation signal may suggest differences between the repetitive DNA sequences of the A and B chromosomes, unless the copy number of the B repetitive sequence on A chromosomes was too low to be detected by FISH. Another possibility is that sequences that are present in low copy number in B chromosome could be under amplified during the probe production, while the high repetitive sequences present in the heterochromatic blocks are overamplified. Although not frequent, findings where B contains sequences not shared with the chromosomes of A complement have been reported in some species, including the mammal *Nyctereutes procyonoides* (Gray, 1834) (Trifonov et al. 2002) and the fish *Alburnus alburnus* (Linnaeus, 1758)(Ziegler et al. 2003). In the vast majority of the cases, sequences found in the B are also shown in A chromosomes, as in the rodent *Apodemus peninsulae* (Thomas, 1907) (Karamysheva et al. 2002) and in the locust *Locusta migratoria* (Linnaeus, 1758) (Teruel et al. 2009). In the plant *Brachyscome dichromosomatica* Carter, 1978, bearing two types of supernumerary chromosomes (Leach et al. 1995; Houben et al. 1997; Houben et al. 2001), some of the repetitive sequences were found only in the B chromosomes, some were present in both B and A chromosomes, and others were present in the B or A chromosomes of other plants of the *Brachycome* genus.

In the investigations on the possible sequence homology of the supernumerary with chromosomes of the closely related *Hypsiboas raniceps*, the FISH with B probe provided a peculiar painting pattern, but a technical artefact was discarded, since a reproducible pattern was obtained in large metaphase samples of two individuals from distinct localities, using hybridisations with 77% stringency. The observed pattern throughout the chromosomes closely resembles that shown in FISH experiments with retrotransposon probes (Meles et al. 2008; Cioffi et al. 2012), but if we are dealing with transposable elements should be investigated. The presence of transposons on B chromosome is not a novelty, since several studies reported that the Bs mostly contain distinct types of repetitive sequences, including transposable and retrotransposable elements, besides satellite DNA and rDNA (review in Camacho et al. 2005). It is surprising that the repetitive sequences found in B are absent on A chromosomes of *Hypsiboas albopunctatus*, but are abundant in the chromosomes of *Hypsiboas raniceps*. The reverse hybridisation confirms that some sequences are actually shared by the B and the regular chromosomes of *Hypsiboas raniceps*. In *Hypsiboas albopunctatus*, these sequences are accumulated in the heterochromatin of the B, whereas in *Hypsiboas raniceps*, they are dispersed throughout the euchromatin of the chromosomes. The supernumerary of *Hypsiboas albopunctatus* is heterogeneous in content, as it was revealed by replication bands and the reverse hybridisation, confirming our previous finding with C banding and DAPI staining (Gruber et al. 2007). One of the B repetitive sequences is shared with *Hypsiboas raniceps* and seems to correspond to a transposable element.

The present analysis using B probe, allowing the precise identification of the B, confirmed it is a univalent, not pairing with any chromosome of the A complement in metaphase I cells of *Hypsiboas albopunctatus*, and its occasional elimination as micronuclei in spermatogenic cells. The occurrence of the B only in about 29% of the 573 spermatids reinforced that the supernumerary segregation is lower than mendelian ratio, at least in males. May be this would explain the unaltered occurrence of one B per individual in the population of Rio Claro, from our first report until now. It is interesting to remark that the *Hypsiboas albopunctatus* populations of Argentina presented accumulation mechanism, that is usually observed for Bs. While in this species the maximum number was three Bs per individual, in the hylid *Gastrotheca espeletia* Duellman & Hillis, 1987, Schmid et al. (2002) observed that the three types of B chromosomes were found from one up to nine Bs per individual. Certainly, further analyses are still recommended for better understanding of the B chromosome transmission during the meiosis in *Hypsiboas albopunctatus*, and the possible absence of B accumulation during meiosis.

Based on our painting results, is very tempting to suggest an interespecific origin of the B, but this may be a premature conclusion, since the distribution of repetitive sequences on B and A chromosomes can be unequal (Montiel et al. 2012), and we should also consider that the dynamics of repetitive sequences suffers from the influence of diverse factors (Biémont and Vieira 2005). Furthermore, phylogenetic data of Faivovich et al. (2005), Wiens et al. (2010), and Pyron and Wiens (2011) show that, despite of the differences in the diploid number, *Hypsiboas albopunctatus* and *Hypsiboas raniceps* are in the same clade in the phylogenetic tree of *Hypsiboas* genus, and they are included in the same *Hypsiboas albopunctatus* phenetic group. Therefore it is important to consider that both species are closely related and probably they inherited diverse types of DNA from a common ancestor. The origin of B chromosome of *Hypsiboas albopunctatus* remains unsolved and approaches, like the investigation of whole sequencing of the supernumerary DNA, would provide further data to shed light on this question.
